# Gynecologists

**Published:** 1879-12

**Authors:** 


Can any one account for the tendency there exists among Gynecologists to the parting of the name in the middle? For instance, we have T. Gaillard Thomas, T. Spencer Wells, J. Marion Sims Duncan, T. Addison Emmett, C. Henri Leonard, A. Reeves Jackson, etc. Can it be that intimacy with the ladies begets vanity?—*Mich. Med. News.*

